# Blood thicker than water: kinship, disease prevalence and group size drive divergent patterns of infection risk in a social mammal

**DOI:** 10.1098/rspb.2016.0798

**Published:** 2016-07-27

**Authors:** Clare H. Benton, Richard J. Delahay, Andrew Robertson, Robbie A. McDonald, Alastair J. Wilson, Terry A. Burke, Dave Hodgson

**Affiliations:** 1 Animal and Plant Health Agency, National Wildlife Management Centre, Woodchester Park, Gloucestershire, UK; 2 Environment and Sustainability Institute, University of Exeter, Penryn Campus, Cornwall, UK; 3 University of Sheffield, Western Bank, Sheffield, UK; 4 Centre for Ecology and Conservation, College of Life and Environmental Sciences, University of Exeter, Penryn Campus, Cornwall, UK

**Keywords:** social structure, kin structure, infection risk, bovine tuberculosis, European badger, relatedness

## Abstract

The importance of social- and kin-structuring of populations for the transmission of wildlife disease is widely assumed but poorly described. Social structure can help dilute risks of transmission for group members, and is relatively easy to measure, but kin-association represents a further level of population sub-structure that is harder to measure, particularly when association behaviours happen underground. Here, using epidemiological and molecular genetic data from a wild, high-density population of the European badger (*Meles meles*), we quantify the risks of infection with *Mycobacterium bovis* (the causative agent of tuberculosis) in cubs. The risk declines with increasing size of its social group, but this net dilution effect conceals divergent patterns of infection risk. Cubs only enjoy reduced risk when social groups have a higher proportion of test-negative individuals. Cubs suffer higher infection risk in social groups containing resident infectious adults, and these risks are exaggerated when cubs and infectious adults are closely related. We further identify key differences in infection risk associated with resident infectious males and females. We link our results to parent–offspring interactions and other kin-biased association, but also consider the possibility that susceptibility to infection is heritable. These patterns of infection risk help to explain the observation of a herd immunity effect in badgers following low-intensity vaccination campaigns. They also reveal kinship and kin-association to be important, and often hidden, drivers of disease transmission in social mammals.

## 1. Background

Understanding disease transmission within wildlife populations has important applications in the fields of emerging zoonotic diseases [1,2], biodiversity conservation [3] and livestock health [4]. Increasingly, the importance of behavioural heterogeneity and social structure on disease transmission between individuals is being recognized, with these individual level differences scaling up to determine infection dynamics at the population scale [5]. However, in wild populations, capturing these individual behavioural differences and quantifying the resultant effects on disease transmission is challenging, particularly when behavioural associations happen out of sight, e.g. in underground setts.

Heterogeneity in individual transmission rates, defined as variability in the contribution of individual hosts to overall rates of pathogen spread [6], is a key driver of disease dynamics. Several studies have demonstrated a relationship between individual contact dynamics and the transmission of infectious diseases (see review in [5]). For example, an individual's position within a socially structured population may influence the likelihood of it becoming infected [7], as demonstrated in social animals such as meerkats [8]. Certain ‘super-spreader’ individuals within a population may contribute to a disproportionate number of secondary infections [9], owing to a particular behavioural or biological trait or their position within a social network. Kin-biased social behaviours have been demonstrated in a variety of species [10,11]. These can include a wide range of behaviours, such as parental care of young, mutual grooming [12], foraging [13] and helping to raise young in the case of cooperative breeders [14]. This enhanced contact between related individuals is likely to have important implications for disease transmission, as these kin-biased social behaviours afford potential opportunities for pathogen transfer. Generally kin structure, defined as the spatial aggregation of related individuals [15], is proposed to increase individual disease transmission risk in directly transmitted pathogens [16], because transmission rates are expected to be higher between related individuals than between non-related individuals (e.g. Canine Distemper Virus in raccoon populations [16] and Chronic Wasting Disease in white-tailed deer [17]). A greater understanding of heterogeneities in individual disease risk could help to inform management interventions and improve the estimation of parameters in epidemiological models to facilitate more ecologically realistic simulations and predictions [18].

Bovine tuberculosis (TB) remains a critical issue in livestock farming in several parts of the world, including the UK. The European badger (*Meles meles*) is the key wildlife reservoir of bovine TB (caused by *Mycobacterium bovis*) in the UK and, as such, has been subjected to a range of control interventions including culling and vaccination, with the aim of reducing disease transmission to cattle populations. However, it is well documented that the social structure typical of moderate to high density, managed and unmanaged badger populations can have a marked impact on the persistence and transmission of TB [19,20]. As badgers live in social groups within defended territories, this can limit population mixing, such that members of different social groups are less likely to come into close contact than members of the same social group. This heterogeneity in contact behaviour is thought to drive the clustered distribution of *M. bovis* infection in badger populations [20,21]. This relationship between population structure and TB dynamics has been implicated in the unexpected outcomes of management interventions to control TB in badgers and cattle, such that reductions in badger density do not result in proportional reductions in TB transmission [19,22–25]. If social structure limits the spread of TB in badger populations, resulting in a naturally aggregated distribution of infection, then disruption of this social structure may carry with it the possibility of enhanced transmission [19]. Social network analysis has suggested that infected badgers occupy a social position within badger populations such that they facilitate transmission of infection between social groups [26].

Within a socially structured population, it is expected that mixing occurs at two scales: ‘local’ mixing, involving high levels of contact between members of the same social group and ‘global’ mixing, involving occasional mixing with individuals outside the social group [7,27]. In badgers, local mixing is likely to increase the risk of infection among cubs born into social groups harbouring infected adults. Furthermore, within the social group a kinship structure will exist, perhaps yielding heterogeneity in contact rates at a finer scale among group members. ‘Pseudo-vertical transmission’, whereby disease transmission occurs via lactation of offspring by infected dams or via the prolonged and repeated periods of close social contact both pre- and post-emergence from the underground sett environment, has been suggested to play an important role in the maintenance of *M. bovis* infection within badger social groups [28,29]. The importance of the social group environment on early life infection risk in badger cubs has been supported by field trials using the now licenced injectable BadgerBCG vaccine [30]: the risk of TB infection in unvaccinated badger cubs decreased significantly as the proportion of vaccinated individuals in their social group increased [31]. Other studies have shown that the presence of infectious females (i.e. those detected as excreting *Mycobacterium bovis*) within a social group helps to predict the incidence of infection in cubs [20,32] consistent with pseudo-vertical transmission. However, to our knowledge no study to date has considered the impact of kin structure within badger social groups on individual infection risk to cubs.

Here, we determine the impact of kinship and infection prevalence in social groups on the infection risk to young badgers present in the social group. We incorporate individual genotype data to account for kin structure within badger social groups, and TB diagnostic tests of adults and cubs to determine infection prevalence and transmission. We predict that cubs born into social groups where resident excretor badgers are present will be at higher risk of testing positive to TB in their first year than cubs born into social groups where excretor badgers are not present, but further that this effect will be greater when resident excretors are related to the cub.

## 2. Material and methods

All data used in these analyses were collected from the long-term capture–mark–recapture study at Woodchester Park in Gloucestershire. Badgers from this study population have been routinely trapped, up to four times a year, since 1976 [33]. Trapped badgers are brought back to a sampling facility, anaesthetized (for full details, see [33]) and a range of clinical samples taken (oesophageal and tracheal aspirates, faeces, urine, swabs of bite wounds or abscesses) for the detection of *M. bovis* by microbiological culture [34]. Blood samples are collected and used for TB diagnostic testing. Diagnosis of infection is made at the individual level, with no reference to other social group members. The use of multiple diagnostic tests to determine disease status in this study helps to address the shortcomings in sensitivity of the tests when used in isolation [35]. Between 1990 and 2005, the Brock ELISA antibody test [36] and the culture of clinical samples were the diagnostic tests used to assign TB status to individual badgers. From 2006 onwards, the Brock ELISA was replaced with the improved Stat-Pak antibody test [37] and the gamma interferon (γ-IFN) test for T-cell responses to *M. bovis* was introduced [38]. The combination of diagnostic tests used provides a biologically meaningful picture of the progression of disease within an individual [39]. It is thought that the cell-mediated response (as measured by the γ-IFN test) is the first line response to *M. bovis* exposure, whereas the serological response (as measured by the ELISA test and StatPak) takes time to develop as infection progresses [39]. Some individuals then go on to become ‘infectious’, characterized by the excretion of *M. bovis* bacteria via various routes [40,41]. Owing to these changes in diagnostic test use, study period was included as a covariate in these analyses, with study period 1 identifying data from 1990 to 2005 and study period 2 identifying data from 2006 to 2011. Culture from clinical samples is the only diagnostic approach that has been used throughout the entire study period.

### (a) Selection of cubs

In order to select a cohort of cubs for this analysis, we selected first year data from the wider population study for badgers first caught as cubs in the population between 1990 and 2011, yielding 1413 cubs for whom genotype data were available. A cub which received a positive test result from any of the diagnostic tests used in their first year was assigned the status ‘test-positive’, whereas a cub with only negative test results in their first year was assigned the status ‘test-negative’. Cubs were assigned to their assumed birth social group, based on the identity of the group in which they were first trapped. We then used R software [42] to associate these cubs with data (disease status in that year, and sex) of adult badgers (more than or equal to 1 year old) trapped in the same social group in the same year. Many individuals were trapped more than once during a calendar year, but each was assigned to just one social group following established assignment rules [43]. For each cub, we collated: number of resident female ‘excretors’ (females from whom at least one *M. bovis* positive culture had been isolated from a clinical sample from a prior trapping event, divided into ‘relatives’ and ‘non-relatives’—defined below); number of resident male ‘excretors’ (males from whom at least one *M. bovis* positive culture had been isolated from a clinical sample from a prior trapping event, divided into ‘relatives’ and ‘non-relatives’); number of resident ‘blood test-positive’ females (females who had at least one positive result to a TB blood test (ELISA, StatPak or γ-IFN) from a prior trapping event, divided into ‘relatives’ and ‘non-relatives’); and number of resident ‘blood test-positive’ males (males who had at least one positive result to a TB blood test (ELISA, StatPak or γ-IFN) from a prior trapping event, divided into ‘relatives’ and ‘non-relatives’).

### (b) Genotyping

On first capture of an individual, a hair sample was routinely taken and stored in 80% ethanol before being submitted for DNA extraction and genotyping [44]. Genotype data were available for animals trapped from 1990 until 2011 inclusive. We used 22 microsatellite markers, each with four to seven alleles, to derive genotypes for 1413 cubs and 470 adults resident in their social group of birth.

### (c) Relatedness

We used the MicroDrop program [45] to impute missing data in the microsatellite dataset, which adjusts for allelic dropout in the genotypes [46]. Deviations from Hardy–Weinberg equilibrium for each of the 22 microsatellite markers were tested on the MicroDrop-corrected dataset using the hwtest function in the ‘adegenet’ package [47]; none was identified. We also used the Bartlett test of homogeneity in the same package to confirm homogeneity of variance among loci (*p* = 0.78). Data from all microsatellite markers were therefore used to calculate a relatedness matrix. We estimated relatedness between cubs and resident adult members of their birth social group using the R package ‘Demerelate’ (v 0.8–1) [48]. Bootstrap iterations were set to 100. Relatedness was calculated using the Queller and Goodnight *r_xy_* relatedness estimator [49]. This provides an unbiased estimate of relatedness based on the population allele frequencies, and ranges from −1 to 1 with negative and positive values indicating lower- and greater-than-average relatedness, respectively [17]. A negative relatedness value indicates that a pair of individuals had a relatedness coefficient lower than the average pairwise relatedness coefficient calculated from the whole genotyped population. Pairs of cubs and resident adults where the relatedness coefficient was more than or equal to 0.25 were assigned the status ‘relatives’ as 0.25 is the relatedness coefficient between half-siblings [50]. Where the relatedness coefficient was less than 0.25, pairs were assigned the status ‘non-relatives’. Potential misclassification rates were estimated based on previous simulations [51] which considered the number of loci used (22) and the average heterozygosity of these loci (0.68). In our dataset, we estimated that 4% of pairs of unrelated individuals may be misclassified as full sibling pairs (full siblings should have an expected relatedness value of 0.5), and 17% of pairs of unrelated individuals may be misclassified as half-sibling pairs. Our ability to distinguish between full siblings and unrelated individuals was therefore high (96%) and half siblings were correctly distinguished from unrelated individuals more than 80% of the time [51]. Previous genetic analyses using the same microsatellite markers on a different high-density badger population indicated that 22 loci were sufficient to obtain reliable relatedness estimates in a population with a mean pairwise difference of less than 0.03 [52]. The mean relatedness estimate for the Woodchester Park population (based on genotypes collected from 2006 to 2011 inclusive) was 0.02.

### (d) Modelling individual infection risk

In order to investigate factors relating to cub infection risk (at a variety of complexities/scales), we carried out three distinct analyses all consisting of generalized linear mixed models constructed via the R package ‘lme4’ (v1.0–5) [53]. In all cases, social group identity and year were included as random effects. Cub infection status was categorized as a binary response variable, with ‘1’ indicating that at least one positive diagnostic test result had been recorded for that individual in year one and ‘0’ indicating only negative test results being recorded. All analyses were performed with individual cub as the sampling unit. Cubs from social groups where genotype data from less than three adults in the group were available were excluded from the analysis, resulting in a dataset of 1362 cubs. We were careful throughout the analysis and interpretation to avoid the term ‘infected’ or ‘uninfected’: issues of test sensitivity mean that some ‘test-negative’ cubs are in fact infected. To the best of our knowledge, the probability of false negative diagnosis is not influenced by phenotypic traits or social group structure; therefore, we assumed that the chance of obtaining false negative results was equal for all infected cubs.

In the first analysis, we investigated the effect of social group size on the risk of each cub testing positive to a diagnostic TB test in their first year, with social group size and study period included as fixed effects. Wald's *χ*^2^-tests were used to assess significance of fixed effects.

To investigate effects of social group composition on the risk of cubs testing positive to a diagnostic TB test in their first year, we regressed cub infection status against the number of individuals test-positive to any of the diagnostic tests in the social group and the number of individuals test-negative to all of the diagnostic tests in the social group (as fixed effects), along with study period. Wald's *χ*^2^-tests were used to assess significance of fixed effects.

Finally, we teased apart the effects of social group composition and relatedness structure on the risk of cubs testing positive to a diagnostic TB test in their first year, using multi-model inference with model averaging. A global mixed effects model included the following fixed effects: the number of resident female excretors (divided into ‘relatives’ and ‘non-relatives’ of each cub), the number of resident male excretors (divided into ‘relatives’ and ‘non-relatives’), the number of resident blood test-positive females (divided into ‘relatives’ and ‘non-relatives’), the number of resident blood test-positive males (divided into ‘relatives’ and ‘non-relatives’) and the number of test-negative group members. Small sample sizes of excretor adults prevented us from using analyses that considered relatedness as a continuous variable [17]. Model averaging was carried out using the ‘MuMIn’ package (v 1.9.13) [54] on the model set generated from the global model, applying a threshold corrected Akaike information criterion (ΔAICc) value of six units, as this is the threshold suggested to be 95% sure that the most parsimonious model is included in the top model set [55,56]. Parameter estimates and their confidence intervals were extracted from the top model set identified through the model averaging procedure. Concerns regarding possible collinearity of the explanatory variables were addressed using correlation testing among all fixed effects in the global model; the mean correlation was 0.06 and the strongest correlation was only 0.36. The explanatory variables did not suffer variance inflation factors greater than 10 and single-term regression models produced parameter estimates that resembled the results of model averaging in terms of sign, size and significance [57].

In order to investigate alternative model structures, we constructed two additional models for comparison with the global model described above. First, to test whether test-negative badgers were differentially affecting cub infection risk, we constructed a fully complex model in which test-negative badgers were disaggregated by relatedness and sex. Second, to test whether sex was adding any information to the model, we constructed a model in which we collapsed effects across sexes throughout the model (i.e. grouping together related culture positive males and females). Both of these models had higher AIC values than the global model described above (fully complex model, ΔAIC = 15, sex removed model, ΔAIC = 7), thus supporting the selection of the global model structure for further study.

## 3. Results

**Figure 1.**
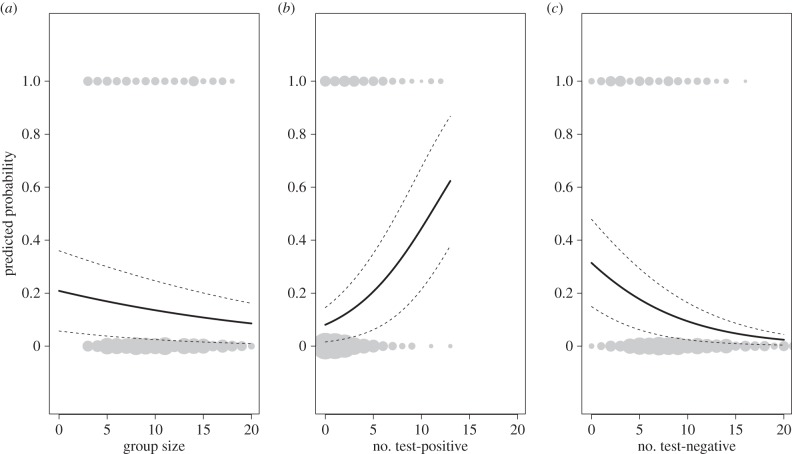
(*a*) Net dilution of the risk of badger cubs testing positive to tests for bovine TB with increasing social group size. (*b*) Increased risk of cubs testing positive within their first year with increasing number of test-positive individuals resident in their social group. (*c*) Reduced risk of cubs testing positive in their first year with increasing number of test-negative individuals resident in their social group. Bold lines indicate line of best fit, dashed lines indicate 95% confidence intervals. Circles summarize the raw data, with the size of symbol proportionally scaled to the number of individuals in that category (smallest point indicates 1 data point, largest point indicates 373 data points).

Of the 1362 cubs included in this analysis, 184 yielded a positive test result within their first year (14%). Summary statistics of social group size and the number of adults in each relatedness—disease category are given in the electronic supplementary material, table S1. In the first analysis, cub risk declined with increasing group size (Wald's 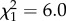, *p* = 0.01), indicating that cubs born into larger social groups were at a lower risk of yielding a positive test result in their first year (figure 1*a*). Study period did not influence the risk of cubs testing positive (Wald's 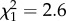, *p* = 0.11). In the second analysis, where group size was elaborated into the number of ‘test-negative’ and ‘test-positive’ individuals present in the cub's natal social group, cub risk increased with increasing numbers of test-positive individuals (Wald's 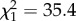, *p* < 0.01; figure 1*b*) but declined with increasing numbers of test-negative individuals (Wald's 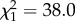, *p* < 0.01; figure 1*c*).

**Figure 2.**
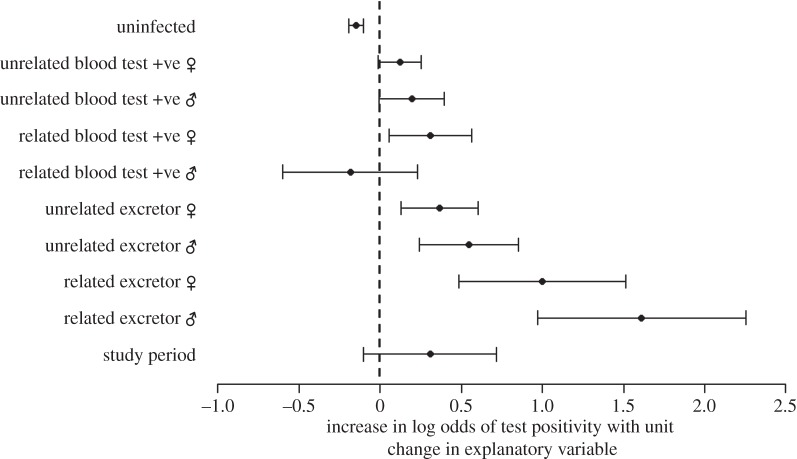
Factors affecting the risk of badger cubs testing positive for bovine TB in their first year (1990–2005). Average model coefficients (log odds) calculated for variables included in the top model set (electronic supplementary material, table S2). Arrows indicate 95% confidence intervals. Model-averaged regression slopes are considered important if they are consistent and directional (i.e. their confidence intervals do not span zero).

**Figure 3.**
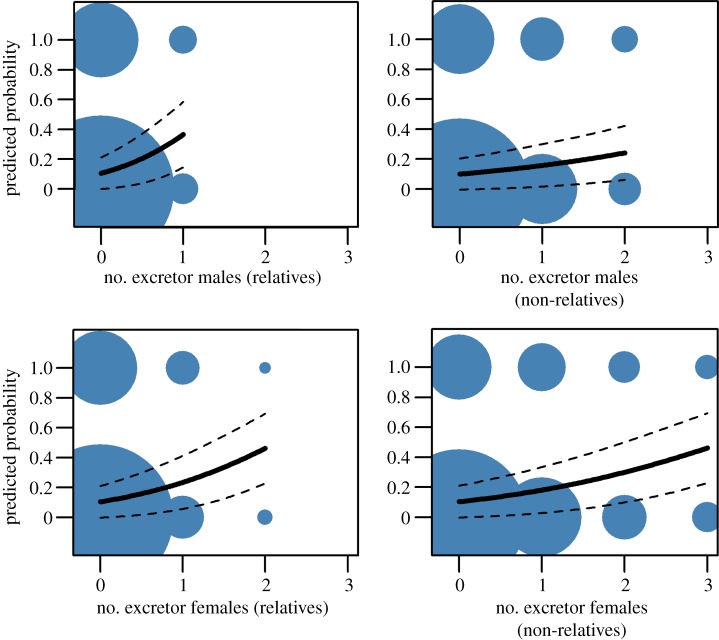
Predicted probability of a cub testing positive for TB in its first year with increasing numbers of excretor relatives and non-relatives resident in its social group. Bold lines indicate the line of best fit; dashed lines indicate 95% confidence intervals. Circles summarize raw data, with the size of symbol proportionally scaled to the number of individuals in the category (smallest point indicates three data points, largest point indicates 1336 data points). (Online version in colour.)

In the final analysis, where test-positive badgers were broken down into the categories described above, model averaging indicated that several variables were important predictors of cub infection risk (electronic supplementary material, table S2). The risk of a cub becoming test-positive in its first year increased most markedly with changes in the number of related excretors of both sexes (figures 2 and 3). The presence of one related male excretor in their birth social group increases the predicted probability of that cub testing positive within their first year by 26%, whereas the presence of a related female excretor increases the probability by 15%. Much lower risks are associated with unrelated male or female excretors (6% and 4%, respectively; barely credibly different from zero (figures 2 and 3)). The probability of test positivity in cubs increased in the presence of blood test-positive female relatives in the social group (electronic supplementary material, figure S1; the presence of one blood test-positive female increases risk by 4%), but was not influenced by blood test-positive male relatives, nor by blood test-positive unrelated individuals of either sex (figure 2).

## 4. Discussion

The findings of this study highlight the potential complexities of transmission dynamics within wild animal populations. When the influence of badger social group size on transmission risks was considered in isolation, we found that cubs born into larger groups were at a lower risk of yielding a positive test result in their first year, indicating net negative density dependence and therefore an important dilution effect on transmission. This is consistent with previous studies in which *M. bovis* prevalence was found to be consistently higher in small social groups [58]. When this simple measure of group size was decomposed according to the test history of resident badgers, the risk of a test-positive result in cubs was positively related to the number of test-positive residents and was only diluted by test-negative residents. This is consistent with the herd immunity effect demonstrated in a vaccinated badger population, whereby the infection risk in unvaccinated badger cubs was reduced where more than a third of their birth social group was vaccinated [31]. Our observation of divergent infection risks, associated with numbers of test-positive versus test-negative individuals, highlights the dangers of relying on population-level metrics (such as host density) to reveal transmission dynamics [5], which in reality may be driven by processes operating at a finer scale.

Further complexity was revealed when social group composition was broken down into kin- and non-kin structures. The number of related female badgers in a cub's natal social group that were excreting *M. bovis* bacteria was positively associated with the risk of that cub testing positive during its first year. This is consistent with infection risk driven by kin-biased association, i.e. closer, more prolonged or more regular contacts between cubs and female relatives than non-relative female group members. Previous studies within high-density badger populations have indicated that females are more likely to be related to other individuals in their social group [52], perhaps because female badgers are less likely to permanently leave their natal group than males [59,60]. Therefore, a cub may be born into a group where multiple female excretor relatives are present, including their mother and sisters from previous years’ litters. Cubs are born and suckled by their mothers during their first 12 weeks of life [61] and may be particularly susceptible to infection in early life when their immune systems are under-developed, making them vulnerable to high infective doses of *M. bovis* from infectious excretor dams [32]. Behavioural monitoring using radio collars shows that females, including younger and non-breeding females, use main setts more during this period than sub-adult and adult males [62]. Cubs may therefore be exposed to infection, both from their mother and from other female badgers present in the main sett prior to emergence. Following emergence from the sett, which occurs at around eight weeks of age, cubs only spend short periods of time above the ground [61] and will remain closely associated with their mothers after emergence, until they are capable of independent foraging. Above ground, anecdotal evidence exists of non-breeding adult females babysitting [63] and allogrooming cubs, although these behaviours did not appear to be kin-biased [64]. Overall the evidence for alloparental care in badgers is considered to be weak [61]. In addition to excretor females posing a risk to resident cubs, we also demonstrated that the number of female relatives in a social group who yielded a positive result to a serological or γ-IFN test was associated with a slight but significant increase in the risk of resident cubs testing positive in their first year. This was not the case for unrelated females or sero/γ-IFN-positive males. As expected, this risk was far lower than for cubs where related or non-related excretor females were resident, reflecting the particular epidemiological importance of infectious individuals in maintaining infection within the social group.

In contrast to previous work [32], the presence of excretor males in a cub's social group was a greater risk factor for cub infection risk than that of female excretors. This result is somewhat surprising, given our understanding of the greater intensity of cub-female behavioural interactions. Paternal care has not been documented in the European badger [65] and is not supported by observational studies [64]. The primary route of bovine TB (bTB) transmission between badgers is considered to be via the respiratory system, such that close and prolonged contact between individuals in setts may facilitate transmission [62]. Male badgers use more of the underground space than females [66]; therefore, excretor male badgers might be more responsible for contaminating the underground sett environment than female excretors. However, this does not explain the difference in risk presented by related and non-related male badgers. Alternatively, opportunities for disease transmission might be owing to above-ground contact as cubs become integrated into the social group following emergence. An observational study of cub social integration following emergence noted that as cubs matured they spent more time and engaged in play-fights more frequently with adult and sub-adult male group members (and less with female group members) [67]. We do not yet know whether these behaviours are kin-biased.

We have shown that the risk to cubs of acquiring infection depends on within-social group structuring, particularly linked to kin and sex. The patterns we observe are consistent with the ‘herd immunity’ in badger social groups, where the risk of TB infection in unvaccinated badger cubs decreased by nearly 80% when more than a third of the social group were vaccinated against TB [31]. Vaccinating a modest proportion of the adults in a badger social group may protect unvaccinated cubs indirectly by reducing their contact with infected adults. Our results suggest that kinship with vaccinated adults will provide cubs with even greater levels of protection.

We note a possible alternative explanation for the higher risk experienced by cubs who have a culture positive relative in their social group: susceptibility to bTB might be heritable. We know that cattle breeds show differential susceptibility to bTB infection [68], and that heritability of bTB resistance in farmed red deer [69] and of bTB disease outcomes in cattle [70] can be high. No published work is currently available on genetic variation in bTB susceptibility in badgers and other wildlife hosts. As the full pedigree of the Woodchester Park badger population emerges in the near future, it will allow us to tease apart the influence of kin-biased behaviour and heritability in bTB transmission dynamics.

Our findings have clear relevance for the understanding, modelling, prediction and management of disease in socially and kin-structured host populations. Social structure can have major impacts on the success of strategies to manage or control disease prevalence and transmission [19,22–25], and the identification of kinship and disease prevalence as mediators of density-dependent transmission could provide important insights to disease management via targeted vaccination or removal campaigns [71]. Kin structure is often hard to identify, and the behavioural interactions that drive direct transmission of disease are often hidden from observation, but their importance to patterns of disease transmission make the advent of molecular tools for wildlife disease all the more relevant [72].

## 5. Conclusion

We have confirmed the epidemiological importance of infectious individuals in the maintenance and persistence of infection in groups of social mammals. We have demonstrated that kin structure causes within-group heterogeneities in infection risks for cubs, either through kin-biased association favouring disease transmission, heritable susceptibility or a combination of the two. Given that strategies for the management of disease in wild mammal populations can perturb social and kinship structures, these key drivers of disease transmission should be considered during the design and delivery of management strategies in wildlife reservoirs of disease. More generally, our findings highlight the potential for conflicting impacts of density, disease prevalence, and social- and kin-structure, on the transmission of disease. In badgers, blood is thicker than water because kinship with test-positive individuals can counteract the dilution effect of reduced infection risk with increasing group size.

## Supplementary Material

Supplementary Table 1

## Supplementary Material

Supplementary Table 2

## Supplementary Material

Supplementary Figure 1

## Data accessibility

For those interested in collaborating in the use of these data, e-mail. dez.delahay@apha.gsi.gov.uk.
