# Supplementary material for: Blood thicker than water: kinship, disease prevalence and group size drive divergent patterns of infection risk in a social mammal
Source: Proc Biol Sci. 2016 Jul 27;283(1835):20160798. doi: 10.1098/rspb.2016.0798 (PMC4971205; doi:10.1098/rspb.2016.0798)
Supplement: Supplementary Table 1 [file rspb20160798supp1.pdf]

Supplementary Table 1. Summary statistics of social group sizes and numbers of adults in disease-relatedness categories.

Category	Mean Value (SD)	Median Value	Range
Social Group Size	11 (5.3)	10	3-31
No. of cubs	3 (2.2)	3	1-12
Proportion of test positive cubs	0.15 (0.3)	0	0-1
		**Proportion of cubs with at least 1 positive in category**	
Related Residents			
Culture positive ♀	0.04 (0.2)	0.04	0-2
Culture positive ♂	0.02 (0.1)	0.02	0-2
Blood test positive ♀	0.13 (0.4)	0.1	0-4
Blood test positive ♂	0.09 (0.3)	0.07	0-3
Test negative ♀	0.7 (1.1)	0.4	0-8
Test negative ♂	0.5 (0.9)	0.4	0-6
Unrelated Residents			
Culture positive ♀	0.31 (0.6)	0.2	0-4
Culture positive ♂	0.17 (0.4)	0.15	0-3

Supplementary Table 1. Summary statistics of social group sizes and numbers of adults in disease-relatedness categories.

Blood test positive ♀	0.75 (1.2)	0.4	0-6
Blood test positive ♂	0.44 (0.7)	0.3	0-4
Test negative ♀	4.6 (3.4)	0.9	0-18
Test negative ♂	3.2 (2.3)	0.9	0-11
