# Supplementary material for: Blood thicker than water: kinship, disease prevalence and group size drive divergent patterns of infection risk in a social mammal
Source: Proc Biol Sci. 2016 Jul 27;283(1835):20160798. doi: 10.1098/rspb.2016.0798 (PMC4971205; doi:10.1098/rspb.2016.0798)
Supplement: Supplementary Table 2 [file rspb20160798supp2.pdf]

Supplementary Table 2. Details of top 10 models with a ΔAICc<6 predicting the odds of cubs testing TB positive in their first year (1). Social group and Study Year were included as random effects. 'Blood test positive' indicates individuals who yielded a positive test result to either the ELISA, StatPak or gamma interferon diagnostic test. Each row in the table indicates a model, with a + indicating the inclusion of a given variable within each model. Degrees of freedom, ΔAICc, model weight and R^2 values are also included for each model. Marginal R^2 (R^2M) represents the variance explained by fixed factors and Conditional R^2 (R^2C) represents the variance explained by both fixed and random factors [54].

Model	Related Excretor Males	Non-Related Excretor Males	Related Excretor Females	Non-Related Excretor Females	Related blood test positive Females	Non-Related blood test positive Females	Related blood test positive Males	Non-related blood test positive Males	Uninfected	Study Period	df	ΔAIC_C	ω_i	R^2C	R^2M
1	+	+	+	+	+	+		+	+	+	12	0.00	0.09	0.35	0.28
2	+	+	+	+	+	+		+	+		11	0.21	0.09	0.35	0.28
3	+	+	+	+	+			+	+		10	0.31	0.08	0.36	0.27
4	+	+	+	+	+			+	+	+	11	0.53	0.07	0.35	0.27
5	+	+	+	+	+	+			+		10	0.58	0.07	0.36	0.27
6	+	+	+	+	+	+			+	+	11	0.71	0.07	0.36	0.27
7	+	+	+	+	+	+	+	+	+	+	13	0.93	0.06	0.35	0.28
8	+	+	+	+	+	+	+	+	+		12	1.37	0.05	0.35	0.27
9	+	+	+	+	+	+	+		+	+	12	1.52	0.04	0.36	0.27
10	+	+	+	+	+	+	+		+		11	1.64	0.04	0.36	0.27
