# Supplementary figures and images for: Blood thicker than water: kinship, disease prevalence and group size drive divergent patterns of infection risk in a social mammal
Source: Proc Biol Sci. 2016 Jul 27;283(1835):20160798. doi: 10.1098/rspb.2016.0798 (PMC4971205; doi:10.1098/rspb.2016.0798)

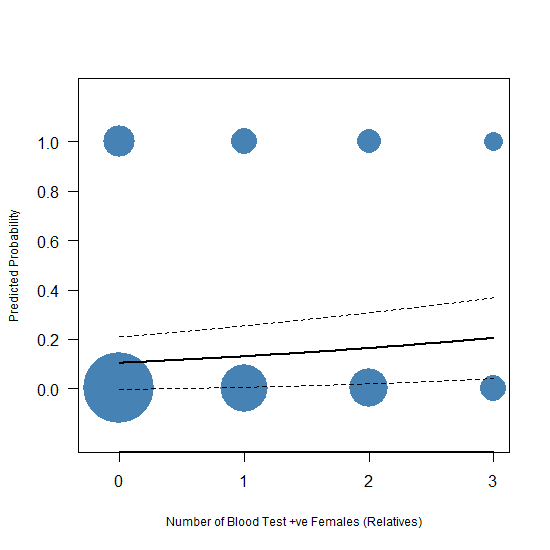

Supplement: Supplementary Figure 1 [file rspb20160798supp3.tiff]
